# Protein Glycosylation in *Aspergillus fumigatus* Is Essential for Cell Wall Synthesis and Serves as a Promising Model of Multicellular Eukaryotic Development

**DOI:** 10.1155/2012/654251

**Published:** 2011-09-28

**Authors:** Cheng Jin

**Affiliations:** Key Laboratory of Systematic Mycology and Lichenology, Institute of Microbiology, Chinese Academy of Sciences, Beijing 100101, China

## Abstract

Glycosylation is a conserved posttranslational modification that is found in all eukaryotes, which helps generate proteins with multiple functions. Our knowledge of glycosylation mainly comes from the investigation of the yeast *Saccharomyces cerevisiae* and mammalian cells. However, during the last decade, glycosylation in the human pathogenic mold *Aspergillus fumigatus* has drawn significant attention. It has been revealed that glycosylation in *A. fumigatus* is crucial for its growth, cell wall synthesis, and development and that the process is more complicated than that found in the budding yeast *S. cerevisiae*. The present paper implies that the investigation of glycosylation in *A. fumigatus* is not only vital for elucidating the mechanism of fungal cell wall synthesis, which will benefit the design of new antifungal therapies, but also helps to understand the role of protein glycosylation in the development of multicellular eukaryotes. This paper describes the advances in functional analysis of protein glycosylation in *A. fumigatus*.

## 1. Introduction

The *Aspergilli* are filamentous fungi, which are multicellular eukaryotes with a relatively simple life cycle. Over 200 species have been classified in the genus *Aspergillus*. Many of them have long been used in food production, industrial fermentation, and agriculture. On the other hand, a few, such as *A. fumigatus*, *A. flavus*, *A. niger*, *A. nidulans,* and *A. terreus*, are opportunistic fungal pathogens, causing life-threatening invasive aspergillosis (IA) in immunosuppressed patients [1, 2], in which *A. fumigatus* is the predominant pathogen [3–5]. The crude mortality for IA is 60–90% and remains around 29–42% even when treatment is given [6]. The main reasons for patient death are late diagnosis and the low efficiency of the drug therapies available to treat IA. 

The fungal cell wall is a protective physical barrier against adverse environmental conditions. The fungal cell wall is a unique organ not found in mammalian cells. It maintains cell shape and provides osmotic protection [7, 8] and has therefore been recognized for a long time as an ideal drug target. Indeed, several cell-wall-targeted drugs, such as echinocandins, caspofungin, micafungin, and anidulafungin, have been introduced as therapies. For example, echinocandins, which inhibit synthesis of *β*-1,3-glucan, a crucial component of the cell wall are effective in the treatment of invasive fungal infections including IA [9]. Unfortunately, the echinocandins also trigger an increase of chitin [9, 10], which partially compensates for the loss of *β*-1,3-glucan and reduces the efficiency of treatment due to the complicated mechanism of cell wall biogenesis in *A. fumigatus*. Therefore, a more profound understanding of the mechanisms of cell wall biosynthesis in *A. fumigatus* would help to improve the efficiency of drug therapies, especially for drugs which target the cell wall.

The cell wall of *A. fumigatus *is composed of a unique *β*-1,3/1,4-glucan skeleton with chitin and galactomannan covalently linked to the nonreducing ends of *β*-1,3-glucan. The cell wall is mainly coated with GPI proteins, which contain N- and O-glycans [11, 12]. While there is no doubt that glycosylation is involved in cell wall organization, the functional importance of protein glycosylation in cell wall organization has, until recently, remained poorly understood. However, during the past few years, it has become increasingly evident that glycosylation is vital for cell wall synthesis and thus vital for growth and morphology of *A. fumigatus*. 

Basically, all eukaryotes possess three types of protein glycosylation, N-glycosylation of asparagine residues, O-glycosylation of threonine and serine residues, and glycosylphosphatidylinositol-anchoring (GPI-anchoring) of the C-terminus of some proteins. Humans lacking individual glycosyltransferases suffer from severe congenital diseases, known as carbohydrate-deficient glycoprotein syndromes (CDGs) [12–14]. Clearly, the sugar components of proteins play a major role in embryonic and postembryonic development of humans as well as of all higher eukaryotes. However, the molecular details leading to CDGs are only vaguely understood. During the past 20 years, the combination of carbohydrate chemistry and biology has developed rapidly. It is now known that carbohydrates play increasingly important roles in regulating the development of higher organisms [15]. However, the mechanism by which carbohydrates play a role in development and disease is still unclear. Our knowledge of protein glycosylation comes mainly from investigation of the model yeast *S. cerevisiae* and of mammalian cells [15]. Although investigation of the model yeast has been very useful in elucidating the biochemical features of protein glycosylation at the cellular level, they cannot reveal the complicated functions of glycosylation in the development of multicellular eukaryotes. Therefore, investigation of glycosylation in the multicellular fungus *A. fumigatus* not only helps understand the mechanism of cell wall synthesis in this species but also provides insights into the role of glycosylation in the development of multicellular eukaryotes. This paper concentrates on protein glycosylation in *A. fumigatus*, which will be discussed with respect to the enzymatic pathways involved and their functional importance. Furthermore, the utility of *A. fumigatus* as a model for glycosylation during development of multicellular eukaryotes will be outlined. 

## 2. Cell Wall Organization and Its Compensatory Mechanism in **A. fumigatus**

### 2.1. Cell Wall Organization

The cell wall of *A. fumigatus* is mainly composed of *β*-1,3-glucans that are highly branched with *β*-1,6 linkages. Together they constitute a three-dimensional network with a large number of nonreducing ends, to which chitin, galactomannan, and *β*-1,3/1,4-glucan are covalently anchored [16]. The cell wall is mainly coated with GPI proteins, which contain N- and O-glycans derived primarily from the process of glycosylation [11, 17]. Glycoproteins such as Gel2p and Ecm33p are also involved in cell wall polysaccharide synthesis. Gel2p is a member of a new family of GPI-anchored *β*1,3-glucanosyltransferases. Deletion of *gel2* leads to slower growth, abnormal conidiogenesis, an altered cell wall composition, and reduced virulence [18, 19]. It has been proposed that Gel2p is responsible for the elongation of *β*-1,3-glucan side chains of *β*-1,3/1,6 branched glucan to provide new nonreducing ends. Ecm33p is also involved in maintaining proper cell wall architecture though its function is unknown. Disruption of *ECM33 *results in morphogenetic aberrations such as defects in conidial separation, increase of chitin in conidial cell walls, rapid conidial germination, and increased virulence [20, 21]. It is clear that glycoproteins are directly, as structural components of the cell wall, and indirectly, as enzymes required for cell wall synthesis, involved in maintaining proper cell wall architecture in *A. fumigatus*. However, it remains unclear how glycosylation affects the function of these proteins. 

### 2.2. Cell Wall Integrity (CWI) Signaling Pathway

Increased chitin synthesis has been known as an important compensatory response to cell wall stress both in* S. cerevisiae *and filamentous fungi [22–26]. In *S. cerevisiae*, the cell wall is required to maintain cell shape, which is essential for the formation of a bud and hence cell division. The yeast cell remodels its rigid structure to accommodate cell expansion during vegetative proliferation, mating pheromone-induced morphogenesis, and nutrient-driven filamentation through the cell wall integrity (CWI) signaling pathway. Cell wall defects or damage induces the cells to activate the CWI pathway to survive, and the compensatory mechanism characterized by an increased chitin content is triggered [27]. 

The CWI signaling pathway in *S. cerevisiae* is activated in response to low osmolarity, thermal stress, or mating pheromone and polarized growth. It is comprised of a family of cell surface sensors coupled to the small G-protein Rho1p, which activates the CWI MAPK cascade via protein kinase C (Pkc1p). This signaling cascade activates the expression of genes encoding for cell wall proteins that stabilize the cell wall. Meanwhile, activated Rho1p also activates a set of additional effectors such as Bni1p and Bnr1p formin proteins, Skn7p transcription factor, and the Sec3p exocyst component, which regulate a diverse set of processes including *β*-glucan synthesis at the site of cell wall remodeling, gene expression related to cell wall biogenesis, organization of the actin cytoskeleton, and secretory vesicle targeting to the growth site [28]. 

A family of cell surface sensors is responsible for detecting and transmitting the status of the cell wall to Rho1p [28]. These sensor molecules include Wsc1p (Hcs77p/Slg1p) [29–31], Wsc2p and Wsc3p [31], and Mid2p and Mtl1p [32, 33]. Among these cell wall stress sensors, Wsc1p and Mid2p appear to be the most important and serve a partially overlapping role in CWI signaling. The extensive O-mannosylation of Mid2p and Wsc1p is important to their stability [34]. Reduced O-mannosylation leads to incorrect proteolytic processing of these proteins, which in turn results in impaired activation of the PKC1 pathway and finally causes cell death in the absence of osmotic stabilization [35]. More recently, N-glycan is found to be directly involved in Mid2p sensing. It has been shown that both the extent of the N-linked glycan and its distance from the plasma membrane affect Mid2p function. Non-N-glycosylated Mid2p fails to perceive cell wall challenges [36]. These observations demonstrate that N- and O-glycosylation are important for CWI sensing and thus important for cell wall biogenesis and polarized growth in yeast. 

The presence of *A. fumigatus *genes encoding for proteins homologous to the yeast Rho1p, Rho3p, and Cdc42p suggests a similar mechanism for the CWI pathway. Indeed, it has been recently shown that Af*cdc42*/*CDC42*, Af*rho1*/*RHO1*, and Af*rho3*/*RHO3 *are highly expressed in the mutant devoid of Cwh41p (glucosidase I), which suggests an activation of these genes induced by cell wall damage [37]. Also, increased expression and activation of the *A. fumigatus* MpkAp, an ortholog of the *S. cerevisiae* Mpk1p, is also induced by cell wall damage [38, 39]. It is becoming clear that, as in yeast, defects in cell wall integrity also trigger the CWI MAPK cascade in *A. fumigatus*.

On the other hand, in contrast to yeast, little is known about the cell wall stress sensors in *A. fumigatus*. In the last release of the *A. fumigatus* genomic database (http://www.aspergillus.org.uk/indexhome.htm?secure/sequence_info/index.php~main), only one protein (AFUA_5G09020) is annotated as a homologue of the Wsc4p, which does not appear to contribute to CWI signaling in yeast. Therefore, the* A. fumigatus* cell wall stress sensor molecule remains to be identified and investigated. 

## 3. Importance of Glycosylation in **A. fumigatus**

The precursor of all mannose residues found in galactomannan, glycoprotein, and GPI anchor in *A. fumigatus* is GDP-mannose. Therefore, its biosynthesis has drawn special attention. In all eukaryotes, the activation of mannose initiates from formation of mannose 6-phosphate (Man-6-P), which occurs by one of two routes: direct phosphorylation of mannose by hexokinase or interconversion from fructose 6-phosphate (Fru-6-P) via phosphomannose isomerase (PMI), and the latter pathway requires three enzymes: PMI, phosphomannomutase (PMM), and GDP-mannose pyrophosphorylase (GMPP). Fru-6-P is converted to Man-6-P by PMI, and then Man-6-P is converted to mannose 1-phosphate (Man-1-P) by PMM. Subsequently, Man-1-P is ligated with the guanosine 5-triphosphate molecule (GTP) to form GDP-mannose by Man-1-P guanylyltransferase [40–63]. 

The interconversion of Man-6-P and Fru-6-P catalysed by PMI is the first committed step in the synthesis of Man-containing sugar chains and provides a link between glucose metabolism and mannosylation. PMI deficiency is the cause of carbohydrate-deficient glycoprotein syndrome type Ib (CDG-Ib, OMIM 602579) in humans, but the clinical symptoms and aberrant glycosylation can be corrected with dietary mannose supplements [40]. 

Genes encoding for PMIs have been investigated in several fungal species, such as *S. cerevisiae*, *Candida albicans*, *A. nidulans*, and* Cryptococcus neoformans* [48–51]. The *S. cerevisiae* PMI is encoded by the *PMI40 *gene [51]. The *pmi* ^−^ mutant shows a significantly reduced growth rate at high concentrations of mannose. Biochemical and genome-wide analysis reveals that excess mannose leads to an accumulation of Man-6-P, which mainly inhibits the activity of phosphoglucose isomerase (PGI) and thus represses glycolysis, protein biosynthesis, and cell wall biogenesis [52]. 

The *A. nidulans manA1* mutant exhibits abnormal ballooning of hyphal tips and eventually ceases to grow [53]. Disrupted *MAN1* mutant of *C. neoformans* leads to poor capsule formation, reduced polysaccharide secretion, morphological abnormalities, and attenuated virulence [50]. In *A. fumigatus*, PMI activity is essential for viability and plays a central regulatory role in both glycosylation and energy production. Deletion of the *A. fumigatus pmi1* gene leads to uncoupling of the link between energy production and glycosylation and accumulation of Man-6-P, which then results in defects in cell wall integrity, conidiation, and morphology [54]. Although extracellular mannose can rescue the growth of PMI deficient mutants in *A. fumigatus*, both lower and higher concentrations of mannose lead to a reduction in the levels of *α*-glucan in the cell wall and an accumulation of Man-6-P [54]. The phenotypes associated with the mutant under mannose starvation are mainly due to an insufficient supply of GDP-Man required for cell wall synthesis. The abnormal morphology associated with the Δ*pmi1* mutant under mannose-replete conditions is mainly ascribed to an accumulation of Man-6-P, which cannot efficiently enter glycolysis, instead becoming trapped in a cycle of dephosphorylation and rephosphorylation resulting in depletion of intracellular ATP. It should be pointed out that the PMI in *A. fumigatus* mainly catalyzes the conversion of Fru-6-P to Man-6-P, and its binding affinity for Man-6-P is similar to that of yeasts but different from the ones from bacteria or animals (Table 1). This suggests that it may be possible to design a specific inhibitor for fungal PMIs [54].

GMPP is the final enzyme in the pathway generating GDP-mannose. Several GMPPs have been identified and characterized in different species [55–59]. In *S. cerevisiae* and *C. albicans*, GMPP is essential [60, 61], while in *Leishmania mexicana* GMPP is not required for viability [62]. Repression of GMPP in yeast leads to pleiotropic phenotypes including cell lysis, failure of cell separation, impaired budding and hyphal switching, clumping and flocculation, and cell wall defects [61]. Repression of expression of *A. fumigatus* GMPP *srb1*, a homologue of yeast *SRB1*/*PSA1*/*VIG9*, leads to hyphal lysis, a defective cell wall, impaired polarity maintenance, and branching site selection, as well as rapid germination and reduced conidiation. In contrast to yeast, inducible repression of *srb1* expression in *A. fumigatus* does affect the ability to direct polarity establishment and septation [63]. 

These reports imply that mannose activation is specifically crucial for the synthesis and organization of the cell wall and thus essential for survival of fungal species. This further suggests that glycosylation is essential for the viability of pathogenic fungal species such as *A. fumigates,* and inhibitors that specifically block mannose activation in fungi may be potential drugs to treat fungal infections.

## 4. Biosynthesis and Function of N-Glycosylation in **A. fumigatus**

N-glycosylated proteins contain oligosaccharides that are N-glycosidically linked to the *γ*-amido group of asparagine. This type of glycoprotein has been intensively studied in many model systems from yeast to human cells with respect to their structure, biosynthesis, and function [15]. It has been shown that the formation of the highly variable N-linked oligosaccharides is initiated by the assembly of a lipid-linked oligosaccharide Glc_3_Man_9_GlcNAc_2_-PP-Dol by a series of glycosyltransferases located on the cytoplasmic and luminal faces of the ER membrane. The most complete understanding of biosynthesis of the lipid bound precursor has been obtained from *S. cerevisiae* and from mammals. As far as it is known, the corresponding reactions proceed almost identically in other eukaryotes [15]. 

**Figure 1:**
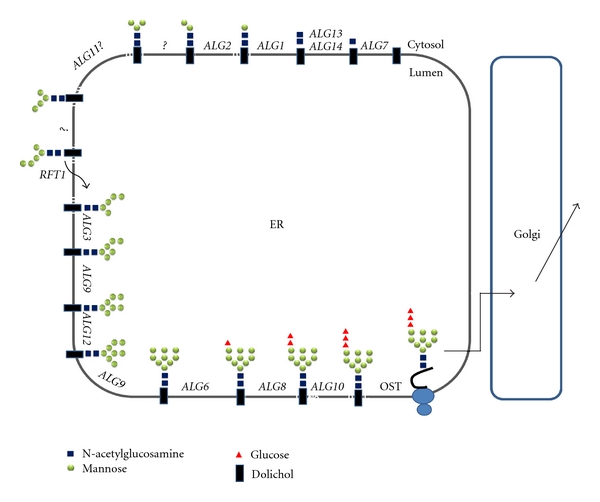
Biosynthesis of the lipid-bound oligosaccharide and transfer of the oligosaccharide to the nascent polypeptide in the endoplasmic reticulum of *S. cerevisiae*. The identified *ALG* genes for the respective glycosylation reactions are indicated. Synthesis starts at the cytoplasmic face with UDP-GlcNAc and GDP-Man as donors. The Man_5_GlcNAc_2_-PP-Dol is then transferred to the luminal side with the help of Rft1 and elongated to the full-length lipid-linked oligosaccharide Glc_3_Man_9_GlcNAc_2_-PP-Dol by using Dol-P-Man and Dol-P-Glc as donors. The oligosaccharide is subsequently transferred to the *γ*-amido group of the asparagine residues within the consensus sequence Asn-X-Ser/Thr of nascent secretory proteins. This reaction is catalyzed by the oligosaccharyltransferase (OST) complex.

Subsequently, the Dol-PP-linked Glc_3_Man_9_GlcNAc_2_ is transferred as a whole to an asparagine residue within an N-X-T/S consensus sequence of a nascent peptide, which is catalyzed by the oligosaccharyltransferase (OST), and then the N-glycosylated proteins are modified in a species-specific manner and transferred through the secretory pathway to the cell surface where they either get exported or anchored to the plasma membrane, to the extracellular matrix, or to the cell wall (Figure 1). 

### 4.1. Initiation of N-Glycosylation

OST is a membrane complex consisting of several subunits. In *S. cerevisiae*, the OST complex consists of at least eight different subunits, including Ost1p, Ost2p, Wbp1, Stt3p, Swp1p, Ost4p, Ost5p, and Ost3p/Ost6p [64–67]. Although the function of each subunit is still unclear, Stt3p is believed to be the catalytic subunit [68–70], and its homologues are found in almost all eukaryotes [71]. The *S. cerevisiae STT3* is an essential gene [72, 73]. It appears that the *A. fumigatus stt3* is also essential as no viable knockout mutant has been recovered [39]. Repression of the *stt3* gene in *A. fumigatus* leads to a severe retardation of growth and a slight defect in cell wall integrity [39]. Further analysis shows that repression of *stt3* upregulates expression of the genes responsible for glucan and chitin synthesis, especially *gel1*, *gel2*, *fskA*, *chsE, *and *chsG*. Indeed, an increase of cell wall mannoprotein and chitin was observed following repression of the *stt3* gene. However, this upregulation of chitin is not accompanied by an activation of the MpkA kinase. Indeed, only the unfolded protein response (UPR) is induced. As the UPR has been shown to be involved in CWI signaling in *A. fumigatus* [74], it is likely that UPR, instead of the MpkA-dependent CWI signaling pathway, is the major compensatory mechanism induced by repression of the N-glycosylation in *A. fumigatus* [39]. 

### 4.2. N-Glycan Processing and Protein Folding Quality Control in ER

Once Glc_3_Man_9_GlcNAc_2_ is transferred to proteins, the N-glycan is processed sequentially in the ER and Golgi. N-Glycan processing is initiated by the removal of the glucose residues catalyzed by ER glucosidase I and glucosidase II. 

In mammalian cells, N-linked glycan plays a decisive role as a quality control (QC) of the folding of secretory proteins, which is composed of calnexin, calreticulin, UDP-glucose:glycoprotein glucosyltransferase (GT) and glucosidase II (GII) and is essential for cellular survival [75–77]. N-glycans initially serve to increase the hydrophilicity of the as-yet-unstructured nascent polypeptides. Subsequently, the two outermost glucose residues of the N-glycan are removed by the sequential action of glucosidase I (GI) and GII to the monoglucosylated form, which is recognized and bound by calnexin, a type I ER membrane lectin, and calreticulin, its soluble relative. For many glycoproteins, the interaction with calnexin or calreticulin slows down the rate of folding but increases efficiency. GII-catalyzed removal of the third glucose residue follows the dissociation of folding substrates from calnexin and is required for release of native polypeptides from the ER and transport to their final destination. The folding sensor GT adds back a terminal glucose to promote reassociation of nonnative polypeptides released from calnexin, thus prolonging their retention in the ER folding environment. Cycles of de-/reglucosylation might be protracted until the polypeptide released from calnexin fulfills quality control requirements. When correct folding is not achieved, an ER-specific N-glycan-dependent pathway of degradation removes the misfolded proteins. When N-glycosylation is inhibited, the most commonly observed effect is the generation of misfolded, aggregated proteins that fail to reach a functional state [75, 76]. 

Before entering the QC system, the outermost glucose residue of the N-glycan is trimmed by ER glucosidase I [78]. A human inherited glucosidase I deficiency has been reported to result in neonatal birth with severe generalized hypotonia and dysmorphic features [79].

Unlike mammalian cells, *S. cerevisiae* lacks a calnexin cycle and GT and only has an effective mannosidase I-dependent ERAD system [80, 81]. The yeast glucosidase I (Cwh1p) is encoded by the *CWH41 *gene [82]. Mutational defects in the *CWH41 *gene cause severe and selective instability of glycoprotein Kre6p, a putative Golgi glucan synthase required for *β*-l,6-glucan synthesis [23, 83, 84]. 

**Figure 2:**
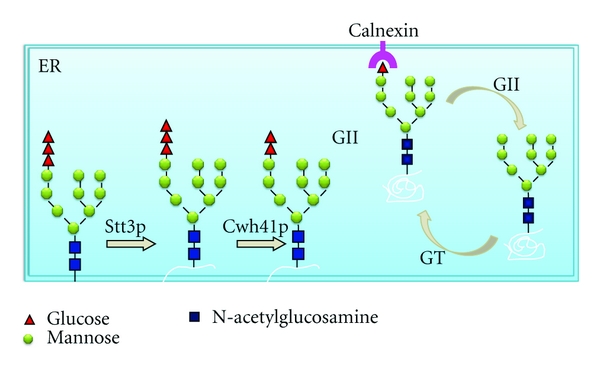
N-glycan-dependent quality control of protein folding in *A. fumigatus*. In *A. fumigatus*, the N-glycan-dependent QC system is composed of calnexin, UDP-glucose: glycoprotein glucosyltransferase (GT) and glucosidase II (GII).

Some filamentous fungi have been proposed to possess N-glycan-dependent QC of glycoprotein folding based on fungal genome sequence data [85]. Recently, evidence that filamentous fungi possess an N-glycan-dependent QC system has been reported in *A. fumigatus* [37, 86]. Indeed, calnexin (AAS68033), glucosidase II, and GT have been annotated in the last release of the *A. fumigatus* genomic database (Figure 2) [87]. Zhang et al. [37] reported that deletion of the *cwh41 *gene in *A. fumigatus *results in defective N-glycan processing of the proteins secreted by *A. fumigatus*. Although Af*cwh41* is not essential for hyphal growth and virulence, a severe reduction in conidial formation, abnormalities of polar growth and septation, and a temperature-sensitive deficiency of cell wall integrity were documented. Also, the genes encoding Rho-type GTPases (Rho-type GTPase/CDC42) were upregulated, which suggests that the CWI pathway was activated in the mutant [37].

### 4.3. N-Glycan Proces sing in the Golgi

**Figure 3:**
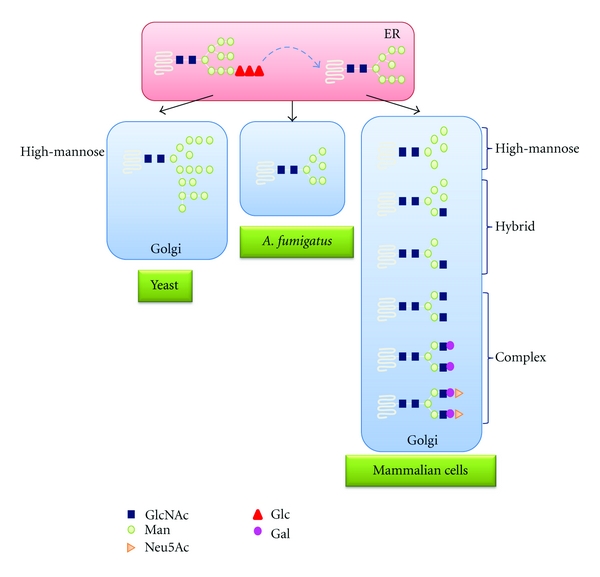
Comparison of N-glycans in yeast, *A. fumigates,* and mammalian cells. After processing by two ER *α*-glucosidases, the N-glycan is further processed by the action of various *α*1,2-mannosidases. In *S. cerevisiae*, a specific ER *α*1,2-mannosidase converts Man_9_GlcNAc_2_ into Man_8_GlcNAc_2_, which is elongated in the Golgi to form an outer chain containing up to 200 residues of mannose. In *A. fumigatus,* the N-glycans on mature glycoprotein are Man_6_GlcNAc_2_. In mammalian cells, Man_9_GlcNAc_2_ is converted to Man_5_GlcNAc_2_ by the action of ER and Golgi *α*-mannosidases, which is the precursor for complex, hybrid, and high-mannose N-glycans.

After processing by two ER *α*-glucosidases, the N-glycan is further processed by the action of various *α*1,2-mannosidases, which can remove one or more of the four *α*1,2-linked mannose residues. In mammalian cells, Man_9_GlcNAc_2_ is converted to Man_5_GlcNAc_2_ by the action of ER and Golgi *α*-mannosidases, which is the precursor for complex, hybrid, and high-mannose N-glycans [88]. In *S. cerevisiae*, a specific ER *α*1,2-mannosidase converts Man_9_GlcNAc_2_ into Man_8_GlcNAc_2_, which is elongated in the Golgi to form an outer chain containing up to 200 residues of mannose [89, 90]. *A. saitoi* and *Trichoderma reesei* have been found to produce N-glycan structures containing five mannose units (Man_5_GlcNAc_2_), suggesting further processing of the Man_9_GlcNAc_2_ precursor [91, 92]. The N-glycans on mature secreted glycoprotein produced by *A. fumigatus* are Man_6_GlcNAc_2_, Man_7_GlcNAc_2_, and Man_8_GlcNAc_2_, in which Man_6_GlcNAc_2_ is the major glycoform [37]. These observations demonstrate that N-glycan synthesis in filamentous fungi seems to differ from that in yeast and is similar to that in higher eukaryotes (Figure 3). Although small N-glycans are commonly found on glycoproteins of *A. fumigatus*, Hex_5-13_HexNAc_2_ glycans on the galactomannoproteins, and Man_5-9_GlcNAc_2_ as well as Gal_*f*1_Man_5-7_GlcNAc_2_ structures on other secreted glycoproteins have been identified in *A. fumigatus* [93, 94]. The enzyme (UDP-Gal*_p_* mutase) required to synthesize the requisite UDP-Gal*_f_* donor has been shown to be an important factor in biosynthesis of the cell wall in* A. fumigatus* [94], while the gene/enzyme responsible for the transfer of Gal*_f_* has not been identified. Recently, the *A. fumigatus* Och1, a key mannosyltransferase for synthesis of elaborated protein N-glycans in yeast, has been identified. Deletion of the *och1* gene results in a reduction of sporulation in the presence of high calcium concentrations. This evidence suggests that polymannosylated N-glycans exist in *A. fumigatus* and certain proteins engaged in sporulation require N-glycan outer chains to be fully functional [95]. 

The *α*-mannosidases have been classified into two groups: Class I and Class II [102, 120, 121]. Class I *α*-mannosidases include ER Man_9_-mannosidase, endomannosidase, and Golgi mannosidase I. Class II *α*-mannosidases include the lysosomal mannosidases, Golgi mannosidase II, yeast vascular mannosidase [98, 99], and ER *α*-mannosidase II [122, 123]. Several Golgi *α*-mannosidases have been cloned and characterized from *Penicillium citrinum* [124, 125]*, A. saitoi* [126]*, A. oryzae* [127]*, T. reesei *[128], and *A. nidulans* [102, 129]. These enzymes are all monomeric with a molecular weight of 50–60 kDa and show the maximal activity in the semiacidic condition (pH 4–6). 

Class I *α*-mannosidase is known to play an important role in the processing of mannose-containing glycans. In *Drosophila melanogaster, *deletion of the Golgi mannosidase I (*MAS-1*) results in viable progeny, and the null organisms synthesize the same range of oligosaccharides as the wild-type ones, albeit at different ratios [130]. In *S. cerevisiae,* disruption of the ER *α*-mannosidase gene does not prevent outer chain synthesis [96]. In the last release of the TIGR database (http://www.aspergillus.org.uk/indexhome.htm?secure/sequence_info/index.php~main) [87], nine *A. fumigatus* genes are annotated to encode *α*-mannosidases, including XP_749038.1, XP_754794.1, XP_751252.1, XP_751819.1, XP_752444.1, XP_752825.1, XP_753592.1, XP_751114.1, and XP_750572.1. Among them, MsdSp (XP_752825.1) has been identified to encode a Class I *α*1, 2-mannosidase and acts on Man_8_GlcNAc_2_ to produce Man_6_GlcNAc_2_. Deletion of the *msdS *gene leads to a defect in N-glycan processing, as well as a reduction of cell wall components (including *α*-glucan, *β*-glucan, mannoprotein, and chitin) and reduced conidiation. Morphological analysis reveals abnormal polarity and septation. However, deletion of the *msdS* has no effect on fungal growth and virulence [97]. 

### 4.4. Degradation of N-Glycan

**Figure 4:**
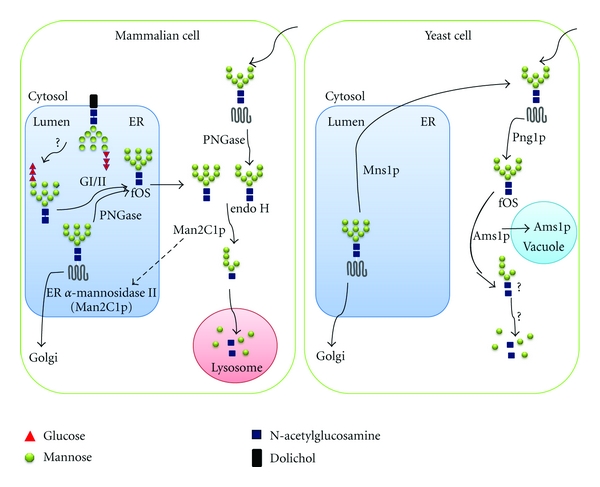
Comparison of free oligosaccharide catabolism in mammalian and yeast cells. Glycoprotein biosynthesis in mammalian cells is accompanied by the generation of free oligosaccharides (fOS) from both OST-mediated hydrolysis of Glc_3_Man_9_GlcNAc_2_-PP-dolichol in the lumen of the ER and peptide N-glycanase (PNGase-) mediated de-N-glycosylation of newly synthesized glycoproteins, which undergo ER associated protein degradation (ERAD), either in the ER or the cytosol. fOS that are liberated in ER can be transported into the cytosol. In the cytosol, fOS are trimmed by an endo-**β**-D-*N-*acetylglucosamine H (endo H-) like enzyme and the *α*-mannosidase Man2C1p to yield Man_5_GlcNAc, which can be imported directly into lysosomes to be degraded. In *S. cerevisiae*, fOS are released from glycoproteins in the cytosol by Png1p, a counterpart of mammalian PNGase. Then the Png1p-generated fOS may be processed in the cytosol by Ams1p, the yeast cytosolic *α*-mannosidase. It should also be noted that no structural studies have been performed on the products that can be generated from Man_8_GlcNAc_2_ by Ams1p, and the ultimate fate of such products remains obscure. On the other hand, two Png1p-independent fOS pools, Man_3_GlcNAc_2_ and Man_8_GlcNAc_2_, are also seen in *S. cerevisiae*. The pool comprising small fOS (Man_3_GlcNAc_2_) appears to be disposed of by unknown enzymes in the vacuole. The pool containing mainly Man_8_GlcNAc_2_ may be generated and disposed of along the secretory pathway.

In mammalian cells, free oligosaccharides (fOS) are generated by OST-mediated hydrolysis of Glc_3_Man_9_GlcNAc_2_-PP-dolichol in the lumen of the ER or peptide N-glycanase (PNGase)-mediated de-N-glycosylation of newly synthesized glycoproteins either in the ER or the cytosol. fOS that are liberated in the lumen of the ER can be transported into the cytosol, where they are trimmed by an endo-**β**-D-*N-*acetylglucosamine H (endo H)-like enzyme and the *α*-mannosidase Man2C1p in order to yield an oligosaccharide, Man_5_GlcNAc, that can be imported directly into lysosomes to be degraded (Figure 4) [122, 123, 131–135]. In humans [136] and cattle [137–139], a deficiency in *α*-mannosidase results in the lethal disease mannosidosis, a rare lysosomal storage disease with a collection of clinical symptoms including progressive mental retardation, impaired hearing, dysostosis multiplex, immune defects, elevation of serum and urinary oligosaccharide levels, and an enlargement of lysosomes in most cell types resulting from the accumulation of undegraded oligosaccharides. 

The rat Man2C1p is involved in oligosaccharide catabolism of misfolded glycoproteins in the lumen of the ER which have been retrotranslocated into the cytoplasm for proteolytic disposal [131–133]. A proteolytically cleaved version of the rat Man2C1p has been found in the lumen of the ER where it is believed to be involved in the early stages of glycoprotein maturation (also called ER *α*-mannosidase II) (Figure 4) [122, 123]. 

The yeast cytosolic *α*-mannosidase Ams1p, a counterpart of Man2C1p, is also involved in the processing of fOS. Since the yeast Png1p is mainly localized to the cytosol, it is proposed that the Png1p-generated fOS may both be generated and processed in the cytosol [100]. The role of the yeast Ams1p is to aid in recycling macromolecular components of the cell under nutrient deprivation [101]. Interestingly, after its synthesis in the cytosol, the Ams1p is translocated into the vacuole by the cytosol-to-vacuole targeting pathway [101], which suggests a common feature shared by the *S. cerevisiae* Ams1p and its mammalian counterparts. However, the yeast Ams1p only participates in recycling or utilizing of oligosaccharide but not in processing of N-glycan (Figure 4) [100]. It should also be noted that no structural studies have been performed on the products that can be generated from Man_8_GlcNAc_2_ by Ams1p, and the ultimate fate of such products remains obscure [135]. On the other hand, two Png1p-independent fOS pools, Man_3_GlcNAc_2_ and Man_8_GlcNAc_2_, are also seen in *S. cerevisiae*. The pool comprising small fOS (Man_3_GlcNAc_2_) appears to be disposed of by unknown enzymes in the vacuole. The pool containing mainly Man_8_GlcNAc_2_ may be generated and disposed of along the secretory pathway [135]. 

Similarly, *A. nidulans α*-mannosidase IIC is also proposed to be involved in oligosaccharide catabolism [102]. Both *A. nidulansα*-mannosidase IIC and *S. cerevisiae* Ams1p are not essential for normal cellular function since disruption of these genes has no visible effect on growth or morphology [98, 99, 102]. 

In contrast to its counterpart in yeast or *A. nidulans*, the *A. fumigatus* Ams1p is required for normal cellular function. Deletion of the *A. fumigatus ams1* leads to a severe defect in conidial formation, especially at a higher temperature. In addition, abnormalities of polarity and septation are associated with the ΔAf*ams1* mutant. These results show that the Af*ams1* gene is required for morphogenesis and cellular function in *A. fumigatus* [103]. The involvement of the Af*ams1* gene in polarized growth demonstrates that the processes involved in fOS regulation are important for *A. fumigatus*. It is likely that the Ams1p is involved in cell wall synthesis and thus polarity through the CWI pathway. Therefore, probably the ΔAf*ams1* mutant could serve as a simple model to investigate the mechanism of *α*-mannosidosis. 

### 4.5. Functions of N-Glycosylation in Cell Wall Synthesis, Morphology, and Polarity

**Figure 5:**
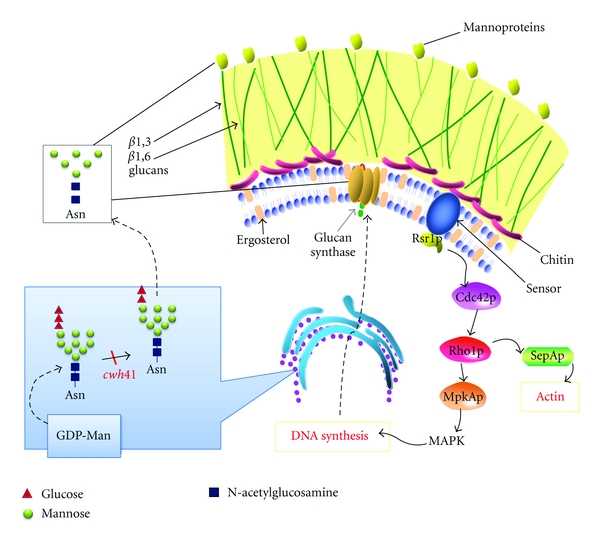
Proposed model of functions of N-glycosylation in *A. fumigatus*. Proteins that are required for cell wall synthesis or cell wall stress sensing require N-glycosylation for their proper folding, localization, and function. Disruption of N-glycosylation in *A. fumigatus* results in misfolding and degradation of these proteins, which thus causes cell wall defects and then leads to the activation of the Rho-type GTPases (Rsr1p/Bud1p-Cdc42p-Rho1p-) mediated CWI pathway to compensate for cell wall defects. Meanwhile activation of the CDC42 in the CWI pathway also activates SepA, an upstream component of actin rearrangement, leading to abnormalities in polarized growth.

Functional analyses of the genes required for N-glycosylation reveal that protein N-glycosylation is important for cell wall synthesis, morphogenesis, and polarized growth in *A. fumigatus*. 2-D gel analysis reveals that deletion of the *cwh41* gene encoding glucosidase I in *A. fumigatus* leads to ER stress, which induces overexpression of HSP70 and calnexin chaperone and activates the ERAD. Meanwhile, the proteins required for actin rearrangement are found to be underexpressed or missing, which is consistent with the observation of random localization of actin fibers in the mutant [86]. These observations, for the first time, clearly suggest that N-glycosylation contributes to proper folding and trafficking in *A. fumigatus*. It appears that proteins involved in cell wall biosynthesis in *A. fumigatus* are more dependent on the N-glycan-dependent folding system. As in yeast, cell wall defects also trigger the CWI signaling pathway in *A. fumigatus*, which activates downstream effectors that regulate cell wall biogenesis and polarized growth. Zhang et al. [37, 86] have proposed that the proteins required for cell wall synthesis or cell wall stress sensing are substrates of *A. fumigatus* Cwh41p and require glucose trimming for their proper localization and function. Misfolding of these proteins would cause cell wall defects, which then leads to activation of the ERAD and Rho-type GTPases-mediated CWI pathway. Moreover, activation of CDC42 in the CWI pathway also activates SepA, an upstream organizer of actin ring formation at septation sites, and thus causes abnormal polarized growth associated with the Δ*afcwh41* mutant (Figure 5). Although the phenotypes associated with different N-glycosylation mutants vary, the finding that all of these mutants exhibit phenotypes associated with cell wall defects, abnormal polarization, and morphological changes can all be explained by this proposed model. 

Obviously, more investigations are needed to identify and characterize all of the proteins affected by N-glycosylation in *A. fumigatus*. This information would be key to understanding the complex compensatory mechanisms participating in cell wall biosynthesis in *A. fumigatus*, which would serve as a basis to develop new antifungal therapies, as well as help to elucidate the molecular mechanism of human diseases associated with defects in glycosylation. 

## 5. Biosynthesis and Function of O-Glycosylation

O-mannose glycosylated proteins were first discovered in yeast and filamentous fungi, and recently this type of glycoproteins has also been described in mammals. The O-mannosylation most likely occurs in all animals, with the exception of nematodes (e.g., *Caenorhabditis elegans*); it is also not detected in plants (*Arabidopsis thaliana*, *Oryza sativa*). However, it has also been discovered in one bacterial species (*Mycobacterium tuberculosis*) [15]. In mammalian cells, the inner O-linked mannose is elongated with the first addition of a N-acetylglucosamine and then various sugars [140]. In the case of yeast, the O-mannose type carbohydrate chain starts with a serine/threonine-linked mannose, which is extended to an oligomannose chain. In *A. fumigatus*, the O-linked glycans on cell wall mannoproteins are found to be Glc*α*1, 6Man, Gal*fβ*1, 6Man*α*1, 6Man, Gal*fβ*1, 5Gal*fβ*1, 6Man*α*1, 6Man and Gal*fβ*1, 5[Gal*fβ*1,5]_3_ Gal*fβ*1, 6Man [141], while only a single mannose residue was detected on secreted proteins [113]. A further type of protein O-glycosylation, in which a single *β*-O-linked GlcNAc residue is linked to serine and threonine occurs in animals, plants, and filamentous fungi, but not in *S. cerevisiae*. For this type of protein modification, a considerable number of reviews are available [142–144]. Therefore, this type of O-glycosylation is not discussed in this paper.

Protein O-mannosylation is initiated by a family of protein O-mannosyltransferases (PMTs) that are evolutionarily conserved from yeast to human [145, 146]. In *S. cerevisiae *a total of seven PMT family members (ScPmt1–7p) are present [104, 147], which fall into three major groups of homology: (i) Pmt1/5/7, (ii) Pmt2/3/6, and (iii) Pmt4. Genes with significant homology to *PMT*s have been cloned in humans, mice, and *Drosophila *[148–150]. Specific protein substrates that are O-mannosylated by ScPmt1p, ScPmt2p, or ScPmt4p have been described in *S. cerevisiae* [151–153].

In comparison with *S. cerevisiae*, the PMT family is less redundant in higher eukaryotes. In *Drosophila *only two PMT family members are present (*rotated abdomen *and *twisted*) [149, 150]. The same is true for mice and humans (POMT1 and POMT2) [148, 150]. Mutations in human POMT1, a homologue of the yeast Pmt4, cause Walker-Warburg Syndrome (WWS), which is characterized by severe congenital muscular dystrophy, neuronal migration defects, and structural abnormalities of the eye [13]. Targeted deletion of *Pomt1 *in mice results in embryonic lethality due to defects in the formation of the Reichert's membrane, the first basement membrane to form in the embryo [154]. Mutations of the *Drosophila *PMT homologues alter muscle structures and the alignment of adult cuticle [155].

The *PMT* family is crucial for viability, cell wall integrity, and morphogenesis in several fungal species, such as *S. cerevisiae*, *S. pombe*, *C. albicans* and *C. neoformans, A. nidulans*, and *A. fumigatus* [108, 109, 111–115, 145, 156]. In *S. cerevisiae*, single *pmt1 *mutants fail to grow in anaerobic conditions on some media [105]. The *pmt1,2,3-*triple mutants grow only in osmotically stabilized medium, whereas the *pmt1,2,4- *and *pmt2,3,4-*triple mutants are not viable in any conditions, indicating that PMT protein activity is essential in *S. cerevisiae*, although individual genes are dispensable [104]. 

*C. albicans *contains five *PMT *genes. The *pmt1* mutants are viable, but they are defective in undergoing cellular differentiation from yeast to a true hyphal growth form under some conditions [106]. The virulence of the *pmt1 *null mutant is significantly attenuated, which is likely due to reduced O-glycosylation of the* C. albicans *adhesin Als1p [106]. The *pmt1,4*-double mutants are not viable. The *pmt *phenotypes are closely linked to alterations in cell wall components, including cell wall mannoproteins and polysaccharides [107]. 

In *S. pombe *only one member of each PMT subfamily is present, namely, *oma*1^+^, *oma*2^+^, and *oma*4^+^. Deletion of *oma*2^+^, as well as simultaneous deletion of *oma*1^+^ and *oma*4^+^ is lethal. Characterization of the viable *S. pombe oma1*D and *oma4*D single mutants shows that reduced O-mannosylation results in abnormal cell wall and septum formation, therefore severely affecting cell morphology and cell-cell separation [108]. In *C. neoformans*, three *PMT *genes are present. Pmt4p is essential for morphogenesis and virulence [109]. Recently, Willger et al. showed that *PMT2 *is an essential gene, and the double *pmt1pmt4 *deletion is lethal [110].

Filamentous fungi, such as *A. fumigatus*, *A. nidulans*,* Neurospora crassa,* and *Fusarium gramineum*, contain only three *pmt* genes that belong to the PMT1, PMT2, and PMT4 subfamilies, respectively [107, 108, 113]. Each of the PMTs appears to function independently. All single *pmt *mutants in *A. nidulans *are viable but showed reduced growth at elevated temperatures and defects in morphogenesis [111, 112]. The double deletion *pmtA/pmtC* (orthologues of the *PMT2 *and *PMT4*) and *pmtB/pmtC *(orthologues of *PMT1 *and *PMT4*) are synthetically lethal. 

Previously, Zhuo et al. have shown that a single deletion of* A. fumigatus pmt1* results in temperature-sensitive phenotypes [113]. When the *A. fumigatus* Δ*pmt1* mutant was grown on solid complete medium at 37°C, no difference was found between the mutant and the wild type. A strongly retarded growth, however, was observed when this mutant was grown at 42°C and 50°C. This temperature-sensitive phenotype could be complemented by the addition of 1 M sucrose in the media. Further analysis shows that the mannoprotein, *α*-glucan, and chitin in the cell wall of the mutant grown at 37°C are increased, while *β*-glucan is reduced. When the *A. fumigatus* Δ*pmt1* mutant was cultured at 42°C, the *α*-glucan was increased, while the *β*-glucan was decreased, and the mannoprotein and chitin content remained unchanged. Moreover, deficient conidiation and reduced germination have been documented at 42°C [113]. As compared with the *S. cerevisiae pmt1* mutants, the *A. fumigatus* Δ*pmt1* mutant, as well as the *C. albicans* and* S. pombe pmt1* mutants, shows more severe defects in cell wall integrity. This significant phenotype could be explained by the fewer members of *PMT* family presented in *A. fumigatus, C. albicans,* and* S. pombe*. However, in a recent study by Mouyna et al., the *A. fumigatus pmt1* mutant does not show any visible phenotype [115]. In the report by Zhuo et al., the *pmt1* deletion mutant was constructed by replacement of the entire coding region of the *pmt1 *in *A. fumigatus* strain CEA17 (*pyrG* ^−^) with a *pyrG *cassette [113]. Therefore, the genetic background of the *pmt1* null mutant is *pyrG* ^+^ *pmt*1^−^, while in the report by Mouyna et al., the *pmt1 *mutant was constructed by transformation of *A. fumigatus *strain ΔKU80 with a deletion cassette containing the *E. coli *phleomycin phosphotransferase gene (*PHLE*) [115]. Therefore, the major differences may be due to the different genetic background of the strains used in these two reports.

The single *pmt2 *or double *pmt1pmt*4 deletion(s) are lethal [114, 115]. Fang et al. [114] reported that reduced expression of *pmt2* leads to retarded growth, cell wall defects, abnormal polarity, and reduced conidiation; however, no temperature-sensitive growth was found. Interestingly, this is the first time that Pmt2p is revealed to be involved in polarized growth. These observations suggest that *A. fumigatus* Pmt2p is required for cell wall synthesis and morphogenesis and its function is distinct from that of *A. fumigatus* Pmt1p. 

Disruption of *A. fumigatus pmt4 *leads to abnormal mycelial growth, poor conidiation, and abnormal polarity. Although an increased sensitivity to echinocandin, a *β*1,3-glucan synthase inhibitor, was observed in the *A. fumigatus pmt4 *null mutant, glucan synthase activity and *β*1,3-glucan content were not affected [115]. In contrast to its counterpart in *C. albicans *[107], *A. fumigatus pmt4* is not required for full virulence. 

The different functions associated with different *A. fumigatus* PMTs are likely due to their different substrate specificities. Further investigation of the *pmt* mutants will be helpful for understanding their molecular mechanism, which will not only increase our understanding of the function of O-mannosylation in *A. fumigatus*, but also may deepen our understanding of the molecular basis of the human Walker-Warburg Syndrome (WWS) which features mutations in POMT1, a homologue of *A. fumigatus* Pmt4p, and results in a failure of polarized growth during neuronal migration [13]. 

## 6. Biosynthesis and Function of GPI Anchoring in *A. fumigatus*

**Figure 6:**
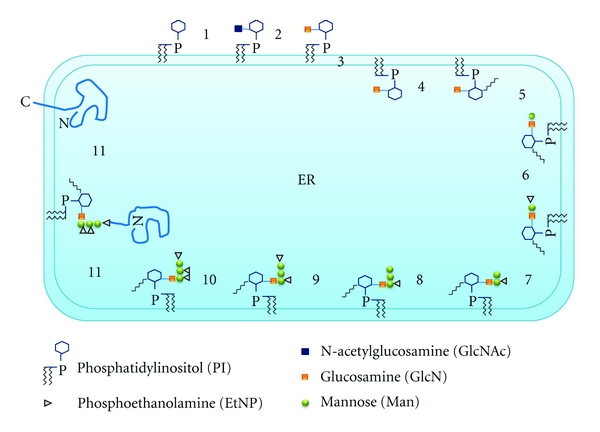
Schematic illustration of GPI biosynthesis in the ER of yeast and mammals. Biosynthesis of the GPI anchor begins at step 1; PI is glycosylated to generate GlcNAc-PI on the cytoplasmic face of the ER. GlcNAc-PI is then de-N-acetylated (step 2) to yield GlcN-PI. GlcN-PI is flipped (step 3) into the lumenal leaflet of the ER, where it is inositol acylated (step 4), inositol mannosylated, and modified by EtNP (steps 5–10). The EtNP-capped GPIs are attached (step 11) to ER-translocated proteins displaying a C-terminal GPI signal sequence. Step  11 is catalyzed by GPI transamidase.

GPI anchoring is a conserved glycosylation process in eukaryotes, which enables many cell surface proteins such as cell surface enzymes, receptors, and adhesion molecules to be covalently anchored to the cell membrane [157]. The core structure of the GPI anchor consists of a lipid group, myoinositol, glucosamine, several mannose residues, and a phosphoethanolamine group, which ultimately connects the GPI anchor to the protein via an amide bond. Although the number of mannose groups and the position of side-chains on the GPI anchors vary widely between species, a common core structure of EtNMan_3_GlcN-PI is conserved in all GPI-anchored proteins found in protozoa, yeast, plants, and mammals (Figure 6). 

GPI anchoring is not essential in mammals at a cellular level as several GPI-deficient cell lines have been established [158]. However, an acquired GPI-anchoring deficiency in haematopoietic stem cells causes paroxysmal nocturnal haemoglobinuria [14], a rare but serious human disease. Also an overexpression of PIG-P, a protein of unknown function required for GPI anchor synthesis, has been noted in fetal Down syndrome brain [159]. In contrast to mammals, GPI anchor synthesis is essential in *S. cerevisiae* [116]. In *S. cerevisiae*, many GPI-anchored proteins are known to be involved in morphogenesis and cell wall organization. Two types of functions have been assigned to these proteins depending on their localization [160]. One type is the GPI-mannoproteins covalently linked to cell wall *β*-1,6-glucan which play important biological functions in filamentation, mating, flocculation, or adhesion to the external matrix [161–167]. The second type are the GPI proteins associated with the plasma membrane which possess enzymatic activities able to modify cell wall polymers and are involved in altering cell morphology, such as *β*-glucanase and *β*-glucosyltransferase [168–170]. Recent studies in *A. fumigatus* suggest that at least nine GPI-anchored proteins are common to filamentous fungi and yeast. Five of them are homologues of putative GPI-anchored yeast proteins that have been shown to play a role in cell wall morphogenesis [160].

The GPI anchor is assembled at the ER in multiple steps catalyzed by the concerted actions of approximately 20 proteins [171]. The first step of GPI anchor synthesis is initiated by the transfer of N-acetylglucosamine (GlcNAc) from UDP-GlcNAc to phosphatidylinositol (PI), which is catalyzed by the glycosylphosphatidylinositol-N-acetyl-glucosaminyltransferase (GPI-GnT) complex. The mammalian GPI-GnT complex consists of seven proteins, including PIG-A, PIG-H, PIG-C, PIG-P, GPI1, PIG-Y, and DPM2 [172]. All except DPM2 have structural and functional counterparts in *S. cerevisiae*, where they are known as Gpi3p, Gpi15p, Gpi2p, Gpi19p, Gpi1p, and Eri1p, respectively [173]. PIG-A/Gpi3p is believed to possess the catalytic domain because Gpi3p binds a photoactivatable UDP-GlcNAc analog and is a member of glycosyltransferase Family 4 of retaining glycosyltransferases [171]. The roles of the other subunits in the GPI-GnT complex are as yet unclear, but they may mediate regulatory interactions. 

In yeast, GPI anchoring is essential for viability and plays an important role in the biosynthesis and organization of the cell wall. A *gpi3* temperature-sensitive mutant is not viable at 37°C [116, 117]. Similar results have been observed in the filamentous fungus *N. crassa* [118]. In both cases, it is postulated that the introduction of mutations in *GPI3*/*gpig-1* genes allows for minimal level of product function and survival when growing the mutant cells below the restrictive temperatures. However, the mechanism, by which the defect in GPI anchoring leads to a lethal phenotype in these two species, is poorly understood. 

It has been shown that *A. fumigatus* GPI anchors possess five mannose residues with a phosphoethanolamine linked on the first three residues [174, 175]. The *A. fumigatus pig-a* gene, the homologue of the *GPI3*/*pig-A *gene in yeast, has been investigated [119]. Deletion of Δ*afpig-a* results in a phenotype characterized by increased cell lysis. Also, an increased content of *β*-glucan and mannoprotein was observed in the mycelial cell wall of the Δ*afpig-a* mutant. Unlike the temperature-sensitive or conditional lethal phenotype seen in the yeast* GPI3 *mutant, Δ*afpig-a* can survive at temperatures from 30°C to 50°C. Completely blocking GPI anchor synthesis in *A. fumigatus* Δ*afpig-a* leads to cell wall defects, abnormal hyphal growth, rapid conidial germination, and aberrant conidiation. *In vivo *assays reveal that the mutant exhibits reduced virulence in immunocompromised mice. Therefore, the GPI anchor seems not essential for viability, but required for cell wall integrity, morphogenesis and virulence in *A. fumigatus. *Indeed, this is the first report that a deficiency in GPI-anchor synthesis does not lead to a temperature-sensitive or conditional lethal phenotype in microbes, which provides an opportunity to identify the basic function of GPI anchoring in fungi. 

## 7. Outlook

During the past 50 years, proteins and nucleic acids have dominated the field of biology. Carbohydrates remained very much on the sidelines. Since the late 1980's, the enormous advances in the analysis of complex carbohydrates have enabled us to investigate the structure and function of carbohydrates, and the field has developed enormously. It is now known that carbohydrates play very important roles, especially in the regulation of development of higher organisms. However, the mechanisms by which carbohydrates play a role in development and diseases are still poorly understood. Our knowledge of protein glycosylation comes mainly from the investigation of *S. cerevisiae* and mammalian cells. Although investigations of the model yeast and mammalian cells have been very useful in elucidating the biochemical features of protein glycosylation, these investigations at the cellular level cannot reflect the complicated functions of glycosylation in the development of multicellular eukaryotes. Therefore, more model systems have been introduced, such as *Caenorhabditis elegans*, *Drosophila melanogaster*, and mice. However, these model systems are still too complex since deletion of individual glycosyltransferase genes in these systems sometimes leads to fetal death or nonvisible phenotypes. As compared with *S. cerevisiae, C. elegans*, *D. melanogaster*, or mice, *A. fumigatus* seems to be an ideal model for investigation of the function of glycosylation since *A. fumigatus* is a multicellular eukaryote with a relative simple life cycle, in which it undergoes a series of developmental events that require polarized growth. Recent progress shows that *A. fumigatus* has evolved an intact N-glycan-dependent QC system, which is present in mammalian cells but not in yeast. Disruption of either processing or degradation of N-glycan in *A. fumigatus* leads to phenotypes such as cell wall defects and abnormal polarity. 

Based on investigations of *S. cerevisiae* and filamentous fungi, it is likely that glycosylation first evolved to ensure synthesis of the fungal cell wall and only later did the N-glycan-dependent QC system evolve to ensure precisely controlled cell wall synthesis and polarized growth which are important for multicellular development. However, this hypothesis is controversial. Recently, based on investigations of numerous protists and fungi, Banerjee et al. [85] showed that the N-glycan-dependent QC system is functional in *Entamoeba*, *Trichomonas*, *Cryptococcus*, and *S. pombe*, but is not functional in some fungi such as *Giardia *and *Plasmodium*, *Theileria*, *Encephalitozoon, Toxoplasma*, *Cryptosporidium*, and *Tetrahymena*. They proposed that the N-glycan-dependent QC system was likely present in the common ancestor of extant eukaryotes and was secondarily lost from some eukaryotes. For example, the *S. cerevisiae *Kre5 is believed to be the GT ortholog that no longer glucosylates misfolded glycoproteins but is instead thought to be involved in *β*-1,6-glucan synthesis [85]. Of course, the possibility that the *S. cerevisiae *Kre5 is the ancestor of GT cannot be excluded. It remains unclear where is the evolutionary origin of glycosylation, what is the basic function of glycosylation at the early stages of evolution, and how glycosylation is regulated. Definitely, the answers to these questions will enable us to understand the basic function and regulation of glycosylation in the development of multicellular eukaryotes and help to understand more complex functions in higher eukaryotes. On the other hand, the investigation of *A. fumigatus* is also a key to understanding complex compensatory mechanisms of cell wall biosynthesis and may provide a new strategy for drug development.

**Figure 7:**
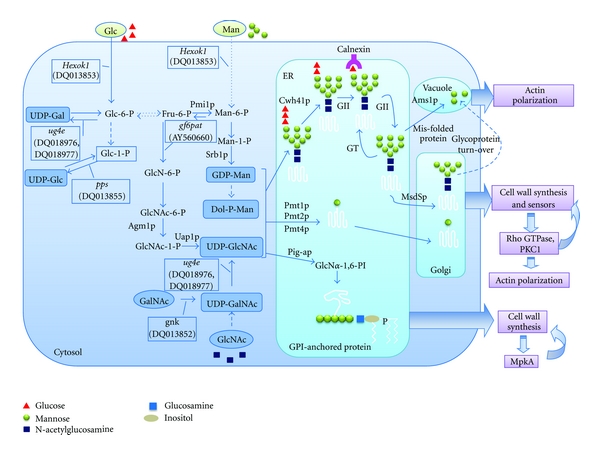
Pathways and functions of protein glycosylation in *A. fumigatus*. In *A. fumigatus*, the activation of mannose initiates from formation of mannose 6-phosphate (Man-6-P), which occurs by one of two routes: direct phosphorylation of mannose by hexokinase or interconversion from fructose 6-phosphate (Fru-6-P), the latter pathway requires three enzymes: phosphomannose isomerase (PMI), phosphomannomutase (PMM), and GDP-mannose pyrophosphorylase (GMPP). Pmi1p (AFUA_1G13280), Sec53p (AFUA_6G06580), and Srb1p (AFUA_6G07620) have been annotated as PMI, PMM, and GMPP, respectively. Functional analyses of Pmi1p and Srb1 imply that mannose activation is specifically crucial for the synthesis and organization of the cell wall and thus essential for survival of *A. fumigatus*. The N-glycosylation is initiated by transfer of Dol-PP-linked Glc_3_Man_9_GlcNAc_2_ to an asparagine residue within an N-X-T/S consensus sequence of a nascent peptide, which is catalyzed by Stt3p (AFUA_8G04430), a putative catalytic subunit of the oligosaccharyltransferase (OST) complex. Subsequently, the N-glycan is processed sequentially in the ER and Golgi. N-Glycan processing is initiated by the removal of the glucose residues catalyzed by ER glucosidase I Cwh41p (AFUA_6G04210) and glucosidase II (AFUA_5G03500) to the monoglucosylated form, which is bound by calnexin (AFUA_4G12850). For many glycoproteins, the interaction with calnexin slows down the rate of folding but increases efficiency. GII-catalyzed removal of the third glucose residue follows the dissociation of folding substrates from calnexin and is required for the release of properly folded proteins from the ER and transport to the Golgi. When correct folding is not achieved, the folding sensor peptide:glucosyltransferase (GT) (AFUA_2G02360) adds back a terminal glucose to promote reassociation of nonnative polypeptides released from calnexin, thus prolonging their retention in the ER folding environment. Cycles of de-/reglucosylation might be protracted until the polypeptide released from calnexin fulfills quality control requirements. Misfolded proteins are removed by an ER-specific N-glycan-dependent pathway of degradation. Ams1p (AFUA_3G08200) has been identified as vacuole *α*-mannosidase that is involved in degradation of N-glycans. Once the native proteins are released from calnexin, the N-glycan is further processed by a yet-unknown ER mannosidase to form Man_8_GlcNAc_2_. Then N-glycosylated proteins are transported into the Golgi, where the N-glycan is trimmed by MsdSp (AFUA_1G14560) to yield Man_6_GlcNAc_2_. When N-glycosylation is inhibited in *A. fumigatus*, the most commonly observed effects are cell wall defects and abnormal polarity, which are likely due to the generation of misfolded, aggregated proteins that are required for cell wall synthesis. The O-mannosylation is catalyzed by Pmt1p (AFUA_3G06450), Pmt2p (AFUA_1G07690), and Pmt4p (AFUA_8G04500), which function independently and are required for cell wall synthesis, thermotolerance, and polarity. Pig-ap (AFUA_1G16950) has been identified as the catalytic subunit of the glycosylphosphatidylinositol-N-acetyl-glucosaminyltransferase (GPI-GnT) complex. GPI anchoring is required for cell wall synthesis, morphology, and virulence.

During the past few years, the framework of the biosynthetic pathways of glycosylation in *A. fumigatus* has been delineated. Functional analyses of some of the genes in this pathway have shown that glycosylation is required for cell wall synthesis, polarity, morphogenesis, and cellular function in *A. fumigatus* (Figure 7 and Table 2). However, a detailed understanding of this pathway remains unknown, such as details regarding the synthesis of the N-glycan precursor, the precise molecular mechanism of N-glycan processing, QC of protein folding, and modification of the GPI anchor. Moreover, the molecular mechanisms by which glycosylation plays a role in morphogenesis and development of *A. fumigatus* are vaguely understood. Therefore, the future direction would be looking for those key proteins that are affected by glycosylation and identifying the signal transduction pathways that link glycosylation and development, through genetic, biochemical, cell biological, and proteomic studies.

## Figures and Tables

**Table 1:** Properties of PMIs from different species.

Species	MW (kD)	*K_m_* for Man-6-P (mM)	*V*_max_ (*μ*mol/min/mg)	Reference
*Aspergillus fumigates*	55	1	753	[54]
*Saccharomyces cerevisiae*	45	0.65	980	[51]
*Escherichia coli*	42	ND	ND	[41]
*Candida albicans*	49	1.24	1200	[42]
human	46.7	0.25	110	[42]
Porcine	ND	0.17	140	[42]
*Salmonella typhimurium*	42.6	1.34	833.3	[43, 44]
*Xanthomonas campestris*	58	2	33.5	[45]
*Pseudomonas aeruginosa*	54	3.03	0.83	[46]
*Burkholderia cepacia*	55	9.01	21	[47]

ND, not detected.

**Table 2:** Summary of the fungal genes studied in glycosylation pathways.

Pathway	Function	Gene	Species	Phenotypes	Reference
		*PMI40*	*S. cerevisiae*	The *pmi* ^−^ mutant only grows on media with exogenous mannose. Excess exogenous mannose leads to an accumulation of Man-6-P, which represses glycolysis, protein biosynthesis, and cell wall biogenesis	[52]
	Phosphomannose isomerase (PMI)	*MAN1*	*C. neoformans*	Disrupted *MAN1* mutant displays poor capsule formation, reduced polysaccharide secretion, morphological abnormalities, and attenuated virulence	[50]
Mannose activation		*manA*	*A. nidulans*	The *manA1* mutant exhibits abnormal ballooning of hyphal tips and eventually ceases to grow.	[53]
		*pmi1*	*A. fumigatus*	Deletion of *pmi1* results in defects in cell wall integrity, conidiation, and morphology. Both lower and higher concentrations of mannose lead to a reduction in the levels of *α*-glucan in the cell wall and an accumulation of Man-6-P in the mutant	[54]
	GDP-mannose pyrophosphorylase (GMPP)	*SRB1*	*S. cerevisiae*	Cell lysis, failure of cell separation, impaired budding and hyphal switching, clumping and flocculation, and cell wall defects	[61]
		*srb1*	*A. fumigatus*	Defective cell wall and impaired polarised growth, as well as rapid germination and reduced conidiation	[63]

	Oligosaccharyltrans- ferase (OST)	*STT3*	*S. cerevisiae*	Essential gene	[72, 73]
		*stt3*	*A. fumigatus*	Repression of the *stt3* gene leads to a severe retardation of growth, a slight defect in cell wall integrity and UPR	[39]
	ER Glucosidase I	*CWH41*	*S. cerevisiae*	Mutational defects in the *CWH41 *gene cause severe and selective instability of glycoprotein Kre6p, a putative Golgi glucan synthase required for *β*-l, 6-glucan synthesis	[23, 83, 84]
		*cwh41*	*A. fumigatus*	Deletion of *cwh41* leads to severe reduction in conidial formation, abnormalities of polar growth, septation and temperature-sensitive deficiency of cell wall integrity.	[37]
N-glycosylation	Golgi mannosidase II	*MSDS*	*S. cerevisiae*	Disruption of t* MSDS* does not prevent outer chain synthesis	[96]
		*msdS*	*A. fumigatus*	Deletion of *msdS *gene leads to a defect in N-glycan processing, as well as a reduction of cell wall components (including *α*-glucan, *β*-glucan, mannoprotein, and chitin), reduced conidiation, abnormal polarity, and septation	[97]
	Cytosolic/vacuolar *α*-mannosidase	*AMS1*	*S. cerevisiae*	The Ams1p is involved in recycling macromolecular components of the cell under nutrient deprivation. Deletion of *AMS1* causes no visible effect on growth or morphology	[98–101]
		*ams1*	*A. nidulans*	oligosaccharide catabolism; no visible effect on growth or morphology	[102]
		*ams1*	*A. fumigatus*	Deletion of *ams1* leads to a severe defect in conidial formation (especially at a higher temperature), abnormalities of polarity, and septation	[103]

		*PMT1 PMT2 PMT3 PMT4 PMT5 PMT6 PMT7*	*S. cerevisiae*	Single *pmt1 *mutants fail to grow in anaerobic conditions on some media. The *pmt1,2,3-*triple mutants grow only in osmotically stabilized medium, whereas the *pmt1,2,4-*and *pmt2,3,4-*triple mutants are not viable in any conditions	[104, 105]
O-glycosylation	mannosyltransferase	*PMT1 PMT2 PMT4 PMT5 PMT6*	*C. albicans*	The *pmt1* mutants are viable, but defective in undergoing cellular differentiation from yeast to a true hyphal growth form under some conditions. The virulence of the *pmt1 *null mutant is significantly attenuated. *pmt1,4*-double mutants are not viable. The *pmt *phenotypes are closely linked to alterations in cell wall components, including cell wall mannoproteins and polysaccharides	[106, 107]
		*oma*1^+^, *oma*2^+^ *oma*4^+^	*S. pombe*	Deletion of *oma*2^+^, as well as simultaneous deletion of *oma*1^+^ and *oma*4^+^, is lethal. The viable *oma1*D and *oma4*D single mutants show abnormal cell wall and septum formation	[108]
		*PMT1 PMT2 PMT4*	*C. neoformans*	Pmt4p is essential for morphogenesis and virulence. *PMT2 *is an essential gene, and the double *pmt1pmt4 *deletion is synthetically lethal	[109, 110]
		*pmtA pmtB pmtC*	*A. nidulans*	All single *pmt *mutants are viable but show reduced growth at elevated temperatures and defects in morphogenesis. Double deletion of *pmtA/pmtC* and *pmtB/pmtC* is lethal	[111, 112]
		*pmt1 pmt2 pmt4*	*A. fumigatus*	Deletion of* pmt1* results in temperature- sensitive phenotypes including retarded growth, cell wall defects, deficient ability of conidiation, and reduced germination	[113–115]
				Single *pmt2 *or double *pmt1pmt*4 deletion(s) are lethal. Repression of *pmt2* leads to retarded growth, cell wall defects, abnormal polarity, and reduced conidiation	
				Disruption of *pmt4 *leads to abnormal mycelial growth, poor conidiation, and abnormal polarity.	

GPI-anchoring	Glycosylphosphatidyl-inositol-N-acetyl- glucosaminyltrans- ferase (GPI-GnT)	*GPI3*	*S. cerevisiae*	A *gpi3* temperature-sensitive mutant is lethal at 37°C	[116, 117]
		*gpig-1*	*N. crassa*	Temperature-sensitive phenotypes	[118]
		*pig-a*	*A. fumigatus*	Deletion of *pig-a* results in a number of phenotypes including increased cell lysis, cell wall defects, abnormal hyphal growth, rapid conidial germination, aberrant conidiation, and reduced virulence	[119]
